# Supplementary figures and images for: Using Implementation Mapping to develop an intervention program to support veterinarians’ adherence to the guideline on Streptococcus suis clinical practice in weaned pigs
Source: PLoS One. 2024 Apr 18;19(4):e0299905. doi: 10.1371/journal.pone.0299905 (PMC11025762; doi:10.1371/journal.pone.0299905)

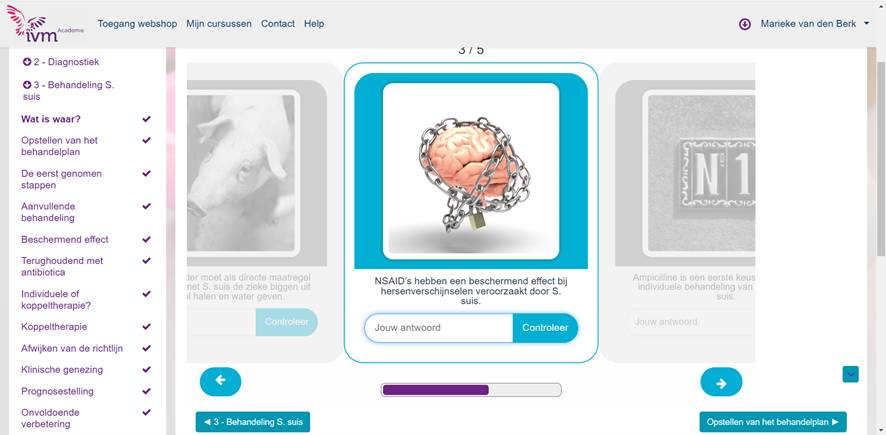

Supplement: S1 Fig — Example method active learning. (TIF) [file pone.0299905.s001.tif]

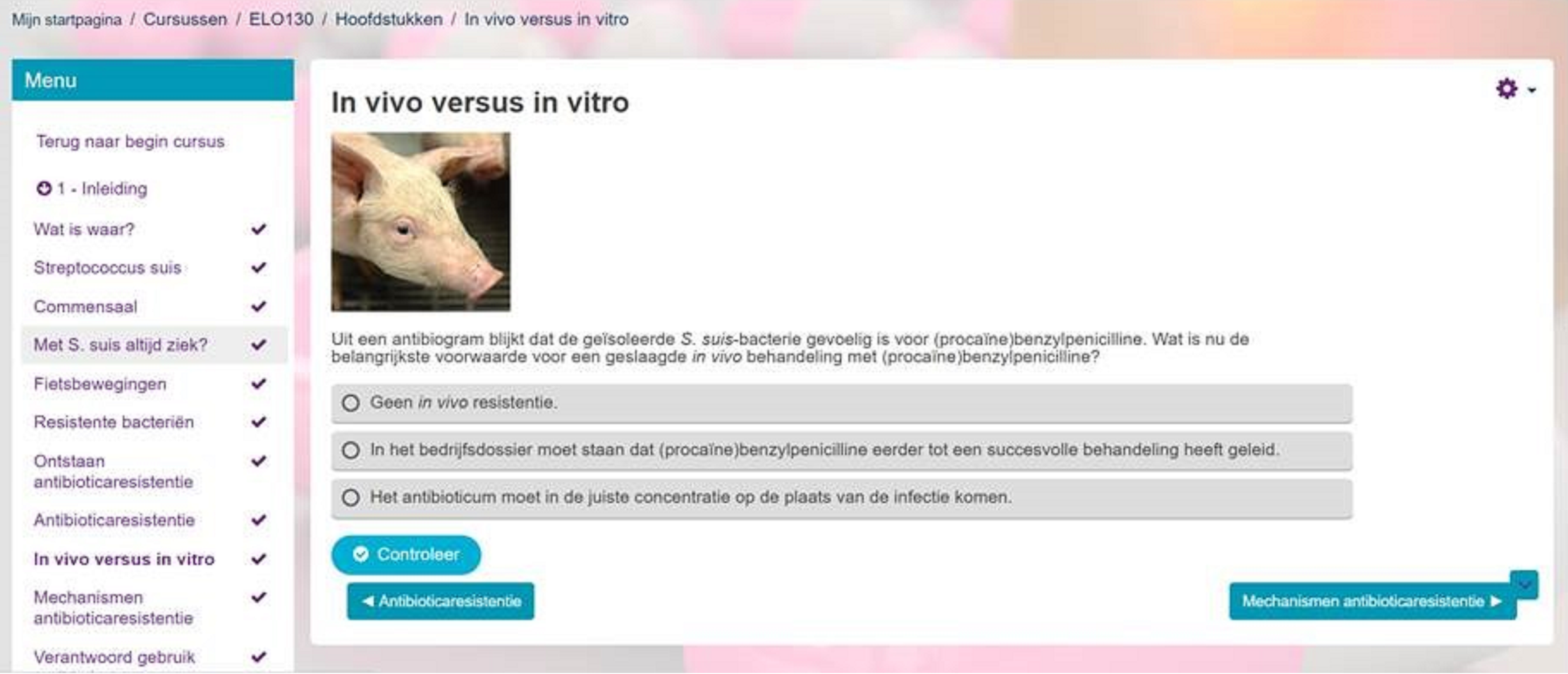

Supplement: S2 Fig — Example method persuasive communication. (TIF) [file pone.0299905.s002.tif]
