# Supplementary material for: Using Implementation Mapping to develop an intervention program to support veterinarians’ adherence to the guideline on Streptococcus suis clinical practice in weaned pigs
Source: PLoS One. 2024 Apr 18;19(4):e0299905. doi: 10.1371/journal.pone.0299905 (PMC11025762; doi:10.1371/journal.pone.0299905)
Supplement: S1 File — S1 Table. Matrix of performance objectives linked to the change objectives. (DOCX) [file pone.0299905.s003.docx]

# Supporting Information

## S1 Matrix of performance objectives linked to the change objectives

| **Performance objective** | | **Knowledge (K)** | **Skills (S)** | **Beliefs about capabilities (B)** | **Beliefs about consequences (C)** |
| --- | --- | --- | --- | --- | --- |
| **1** | **The veterinarian** | The veterinarian … | | | |
|  | **writes complete reports and/or administers at least the following information regarding every *S. suis* problem farm:  a. the findings from clinical examination and an estimation of the number of affected animals; b. the (probable) diagnosis and the potential results of diagnostics; c. the vaccination status;  d. the advice(s) and/or treatment plan; e. motivation for deviation from first choice antimicrobials and the therapy evaluation; f. the number of animals that will be treated and pens and section(s) in which the animals to be treated are located.** | Can state the administrative requirements by law regarding antimicrobial use at swine farms P.1.K.1. | Can quickly create a report, which meets the requirements by law and from inspectors, in a structured way with easy tools P.1.S.1. | Is convinced that (s)he can develop a report with an added value for the farmer P.1.B.1. | Is convinced that a report for the farm is of value for the farm management P.1.C.1. |
|  |  | Can state the requirements for reports as is necessary for the approval of inspectors P.1.K.2. | Can assemble all the information necessary to create a complete report P.1.S.2. |  | Is convinced that making right reports is important for a veterinarian’s professional value P.1.C.2. |
|  |  |  |  |  | Is convinced that farmers read and use the reports in their farm management plan P.1.C.3. |
|  |  |  |  |  | Is convinced of the value of good reports as justification for actions towards third parties P.1.C.4. |
| **2** | **The veterinarian** | The veterinarian … | | | |
|  | **advises the farmer to administer corticosteroids to piglets with nervous disorders caused by *S. suis*.** | Can explain why corticosteroids have a positive effect on the recovery of a piglet with *S. suis* P.2.K.1. | Can convince the farmer of the added value of the use of corticosteroids for *S. suis* piglets P.2.S.1. | Is convinced that (s)he is capable of convincing and educating a farmer about the use of corticosteroids P.2.B.1. | Is convinced that corticosteroids can have an added value for the recovery a piglet with *S. suis* P.2.C.1. |
|  |  | Can tell that routines for the farmer are important and therefore that it is necessary to include corticosteroids in this routine regarding *S. suis* piglets P.2.K.2. |  |  | Expresses how important it is to repeat the advice to use corticosteroids for *S. suis* piglets to the farmer P.2.C.2. |
| **3** | **The Veterinarian** | The veterinarian … | | | |
|  | **advises the removal of sick piglets with a probable diagnosis of meningitis caused by *S. suis* from the flock and providing water to the piglets.** | Can state the direct measures (remove sick piglets with a probable diagnosis of meningitis caused by *S. suis* from the group and provide the piglets with water) from the *S. suis* guideline P.3.K.1. | Can convince the farmer to remove sick piglets from the group P.3.S.1. | Is convinced that (s)he can influence the farmer’s daily routine P.3.B.1. | Is convinced it is important to remove sick piglets from the group to give the piglets the highest chance of recovery P.3.C.1. |
|  |  |  | Can convince the farmer to give sick piglets water P.3.S.2. |  | Is convinced that it is important to give sick piglets water to prevent dehydration P.3.C.2. |
|  |  |  |  |  | Is convinced that it is important to incorporate the following actions in the farmer’s daily routine: removing sick piglets from the group and giving sick piglets water P.3.C.3. |
| **4** |  | The veterinarian … | | | |
|  | **In principle, the veterinarian prescribes first choice antimicrobials for the treatment of *S. suis*.** | Can tell the advantages and disadvantages of all the antimicrobials used for the treatment of *S. suis* P.4.K.1. | Can explain to the farmer the difference between first, second, and third choice antimicrobials P.4.S.1. | Is convinced that (s)he can influence the farmer about which antimicrobial to use (B) P.4.B.1. | Is convinced that first choice antimicrobials are effective for the treatment of *S. suis* P.4.C.1. |
|  |  | Can recall how antimicrobial resistance develops P.4.K.2. | Can explain to the farmer the doses in which veterinary medicals need to be applied to treat *S. suis* piglets (determining piglets’ weight) P.4.S.2. |  | Is convinced that a second choice antimicrobial is not always necessary for a farm to receive good economic results (C) P.4.C.2. |
|  |  | Can tell which guidelines and laws regarding antimicrobials are present in the Netherlands P.4.K.3. | Can convince the farmer to follow her/his advice to treat sick piglets quickly and check them regularly P.4.S.3. |  | Is convinced that the use of first choice antimicrobials is correct to prevent antimicrobial resistance of second choice antimicrobials P.4.C.3. |
| **5** | **The veterinarian** | The veterinarian … | | | |
|  | **prescribes group treatments when 5% or more piglets in the herd/group are affected within 5 days or 4% or more piglets are affected within 24 hours.** | Can recall the percentages from the *S. suis* guideline with regard to group treatment P.5.K.1. | Can educate the farmer in: recognizing the first clinical signs of *S. suis* piglets to increase the speed of treatment, the disadvantages of antimicrobial use and the development of resistance P.5.S.1/V. | Is convinced that (s)he can assess what is going on better than the farmer and make the probable diagnosis P.5.B.1. | Is confident that following the advice of the *S. suis* guideline about treatments will reduce antimicrobial use P.5.C.1. |
|  |  | Can recall that the advice in the *S. suis* guideline for group treatments is for affected piglets (not for dead piglets) P.5.K.2. | Can convince the farmer of the importance of checking piglets at least twice a day P.5.S.2/V. |  | Is convinced that it is good for the farmer to start individual treatments instead of group treatment P.5.C.2. |
|  |  |  | Can discuss the Defined Daily Dose Animal with the farmer to make the *S. suis* problem visible and offer veterinary advice P.5.S.3/V. |  | Does not find the statement from the *S. suis* guideline about group treatments negative for piglet welfare P.5.C.3. |
|  |  |  | Can assess how competent the livestock farmer is (e.g., in diagnosing, estimating severity) and provide standardized methods to properly provide daily activities (e.g., Trays with colors/numbers by section) P.5.S.4/V |  | Is convinced that (s)he can find other solutions for *S. suis* outbreaks instead of immediately starting group treatment P.5.C.4. |
| **7** | **The veterinarian** | The veterinarian … | | | |
|  | **advises post-mortem examinations at a first *S. suis* outbreak (at least 2 piglets twice a year, including bacteriological culturing and susceptibility testing). The veterinarian advises structural post-mortem examination (4 times a year, 2 piglets including susceptibility testing) at an existing *S. suis* problem farm.** | Can recall the statements about post-mortem examination in the *S. suis* guideline (K) P.7.K.1. | Can convince farmer (communication skills/right approach per farmer) to arrange a pathological examination (including bacterial examination) and to give up representative piglets for this purpose (two piglets twice a year and in the case of problem farm two piglets four times a year) P.7.S.1./V. | Is convinced that (s)he can influence the decision on whether and how many and which animals are examined post-mortem P.7.B.1. | Is convinced that pathological and bacteriological examination adds value (to confirm diagnosis, choose antimicrobials, and report *S. suis* types) P.7.C.1. |
|  |  | Can tell that, whether the *S. suis* type changes or not, this tells nothing about the sensitivity of the bacterium P.7.K.2. | Can use the *S. suis* guideline in his/ her persuasion technique to the farmer P.7.S.2. |  | Is convinced that bacteriological research is of added value despite the in vivo - in vitro differences P.7.C.2. |
|  |  | Can tell the added value of bacteriological examination P.7.K.3. | Can explain to the farmer that the results of bacteriological examination are not binding if the farm history has a different outcome (as stated in guideline) P.7.S.3./V. |  | Selects a standard-certified lab to do the bacterial examination P.7.C.3. |
|  |  | Can tell the added value of bacteriological examination relative to impression preparation P.7.K.4. | Can convince the farmer that the results of the pathological examination including bacteriological examination remain of added value even if they appeared predictable P.7.S.4./V. |  |  |
| **8** |  | The veterinarian … | | | |
|  | **If autogenous^a^ vaccines are used, the veterinarian recommends that piglets are regularly examined by a laboratory for serotyping of *S. suis*.** | States that it is important to keep examining piglets in order to keep the overview of *S. suis* types in the Netherlands P.8.K.1. | Can convince farmer of the added value of serotyping of *S. suis* P.8.S.1. | Is convinced (s)he can convince the livestock farmer of the added value of continued serotyping P.8.B.1. | Is convinced that regularly examining piglets for serotyping is necessary for the success of auto vaccination P.8.C.1. |
|  |  | Can tell that a change in the *S. suis* type can make the auto-vaccine ineffective P.8.K.2. | Can assess suitability of farmer to use auto-vaccines (need to structurally offer and maintain correct animals, colostrum provision, no other major risks present) P.8.S.2. |  | Is convinced that, despite the cost, serotyping is of added value to prevent later reduced auto-vaccine efficacy P.8.C.2. |
|  |  | Can tell that it is important to examine several piglets because several types are possible (3–10 at start is mentioned) P.8.K.3. | Can educate the farmer to use auto-vaccines correctly P.8.S.3. |  | Is convinced that value should be placed on auto-vaccine results based on numbers (e.g., on Defined Daily Dose Animal and dropout rates) to determine success P.8.C.3. |
|  |  | Can state the laws and regulations regarding auto-vaccination: prescribing veterinarian is responsible for proper application of auto-vaccine (consequences puncture incident, etc,) and that there are risks involved (possible side effects) P.8.K.4. |  |  |  |
|  | **The veterinarian** | The veterinarian … | | | |
| **9** | **advises the euthanasia of (i) piglets with severe brain symptoms and (ii) piglets that deteriorate within 8 hours or do not recovery sufficiently within 48 hours.** | Can give the following advice from the guideline: euthanize piglets with severe brain symptoms and piglets that deteriorate within 8 hours or do not recovery sufficiently within 48 hours P.9.K.1. | Can convince the farmer of the importance of euthanizing the piglet at the appropriate time (within 8 hours, or when no or insufficient recovery occurs within 48 hours) despite costs P.9.S.1. | Is convinced that his/her role is important in how a farmer treats his piglets and when the farmer proceeds to euthanasia P.9.B.1. | Does not want piglets to suffer unbearably P.9.C.1. |
|  |  | Can tell the possible ways of euthanizing piglets P.9.K.2. | Can develop a relationship of trust with the farmer that allows honest discussion of this issue P.9.S.2. |  | Knows in what way (or not) her/his farmers euthanize piglets and can see the farmers’ mortality figures because (s)he is convinced this is important to discuss for animal welfare P.9.C.2. |
|  |  | Can list the systems (e.g. Beter Leven Keurmerk) and requirements regarding sustainability and when euthanasia is advantageous P.9.K.3. | Can explain and educate the farmer about the possible options for euthanasia and the importance of the way being evidence-based, so that the farmer can routinely perform it correctly P.9.S.3./V |  |  |
|  |  | Can tell the laws regarding euthanizing piglets for veterinarians and farmers P.9.K.4. | Can explain to the farmer how to keep a structured record of euthanized piglets P.9.S.4./V. |  |  |
|  | **The veterinarian** | The veterinarian … | | | |
| **10** | **uses the recommendations of the *S. suis* guideline for the treatment of piglets with meningitis and not with arthritis.** | Can say that the *S. suis* guideline was written for piglets with meningitis and that arthritis requires a different approach P.10.K.1. | Can recognize whether the farmer has arthritis or meningitis *S. suis* problems P.10.S.1. | Can convince the farmer to choose a different treatment protocol for *S. suis* meningitis problems than for *S. suis* arthritis problems P.10.B.1. | Is convinced that the consequences of arthritis are different from those of meningitis and believes that both require a different approach P.10.C.1. |
|  | **The veterinarian** | The veterinarian … | | | |
| **11** | **actively searches for *S. suis* risk factors at the farm (if unknown), for which the checklist in the *S. suis* guideline can be used. On the basis of the risk factors identified, the veterinarian gives tailored advice to prevent *S. suis* problems.** | Can list the *S. suis* risk factors as mentioned in the guideline P.11.K.1. | Can convince farmer and employees to find *S. suis* risk factors P.11.S.1. | Is convinced that (s)he can influence *S. suis* problems and can solve *S. suis* problems P.11.B.1. | Is convinced that all *S. suis* problems can be solved if proper measures are applied P.11.C.1. |
|  |  | Can communicate the most current developments/ latest publications regarding *S. suis* P.11.K.2. | Can convince the farmer and his/her employees to follow veterinary advice on an ongoing basis P.11.S.2./V. | Is convinced that (s)he can influence the farmer’s view on the risk factors on his/her farm P.11.B.2. | Is convinced that it is right to identify the bottleneck(s) even if they cannot be solved immediately P.11.C.2. |
|  |  |  | Can convince the farmer to think out-of-the-box and be open to new methods/advice P.11.S.3/V. |  | Is convinced that the farmer can make an incorrect/ incomplete diagnosis and/or identify incorrect risks factors despite his/her experience P.11.C.3. |
|  |  | Can list the likely risk factors for each of her/his farms with *S. suis* problems P.11.K.4. | Can choose to make an autonomous professional decision under pressure from his/her client P.11.S.4. |  |  |
|  |  | Can provide solutions for the most common *S. suis* risk factors as defined in the *S. suis* guideline P.11.K.5 | Can conclude what the main bottlenecks of a farm are and give advice on this P.11.S.5. |  |  |
|  | **The veterinarian** | The veterinarian … | | | |
| **12** | **does not use the *S. suis* guideline for weaned piglets as a checklist to comply with the sanction system of inspectors, but as a helpful tool in advising swine farmers.** | Can tell the differences between guidelines and legislation in enforcement and how they are developed (composition/ procedure working group) P.12.K.1. | Can communicate correctly with inspectors P.12.S.1. | Can value the content of the *S. suis* guideline on correctness P.12.B.1. | Is convinced that the guideline adds value to her/his actions (in the form of reminder/checklist) to convince the farmer P.12.C.1. |
|  |  | Can say that deviation from the guideline is allowed if it is well documented and can explain what inspectors expect from this documentation P.12.K.2. | Can apply the guidelines to convince the farmer P.12.S.2. |  | Is convinced that the guideline can influence the veterinarian's actions and thereby reduce antimicrobial use P.12.C.2. |
|  |  | Can recall the content of the guideline *S. suis* P.12.K.3. | Uses the guidelines as a tool and back-up for on-farm decisions P.12.S.3. |  |  |
|  | **The veterinarian** | The veterinarian … | | | |
| **13** | **ensures that his/her knowledge about *S. suis* and the rules regarding *S. suis* is kept up to date by following regular refresher courses or reading scientific literature on this subject. The veterinarian visibly applies this knowledge in his/her advice.** | Can list the rules and regulations and therefore knows when a colleague does not have the correct information P.13.K.1. | Can discuss and share knowledge with colleagues and manages to deal with feedback from others and is able to give feedback to colleagues in a positive manner P.13.S.1. | Is convinced that colleagues can learn from her/his experiences and knowledge, and vice versa P.13.B.1. | Is convinced that having evidence-based knowledge has an added value for solving *S. suis* problems at farms P.13.C.1. |
|  |  | States that being at the forefront of laws and regulations improves the quality of the final product, making the market value higher compared to other countries P.13.K.2. | Is critical of his/her own diagnoses P.13.S.2. | Believes (s)he adds value to the farm in advising on laws and regulations and how to deal with them in practice P.13.B.2. | Is convinced that his/her colleagues can contribute to solving *S. suis* problems on her/his *S. suis* farms P.13.C.2. |
|  |  | Knows where to find evidence-based sources regarding *S. suis* P.13.K.3. | Can convince, educate, and advise farmer and his/her employees about the value of new developments and laws and regulations P.13.S.3./V. |  | Is convinced of the added value of being at the forefront of laws and regulations compared with other countries P.13.C.3. |
|  |  |  | Can incorporate rules and legislation into daily work, does not violate them, and still sees sufficient freedom in his/her way of working P.13.S.4. |  | Is convinced that, in the long run, following laws and regulations is beneficial to avoid fines (or other problems as a result of negative inspections), for animal welfare, and for public health P.13.C.4. |
|  |  |  | Listens to advice from others (colleagues, feed supplier, etc.) but chooses to make his/her own autonomous professional decision P.13.S.5. |  |  |
|  |  |  | Can educate the farmer about handling feedback from inspectors so that the feedback given can be processed for the next inspection P.13.S.6./V. |  |  |

^a^Inactivated or non-inactivated immunological veterinary medicinal products manufactured from pathogens and antigens obtained from an animal or animals from a holding and used for the treatment of that animal or the animals of that holding in the same locality [28].

## S2 Print screens e-learning

**S2.1 Fig. Print screen e-learning *S. suis* guideline.** Example method active learning.

**S2.2 Fig Print screen e-learning S. suis guideline.** Example method persuasive communication.

## S3 Questions knowledge quiz

1. Which drug is first choice in the individual treatment of meningitis caused by *S. suis*?

- oxytetracycline

- amoxicillin

- procaine benzylpenicillin

- ampicillin

2. The *S. suis* in weaned piglets guideline (2014) describes the policy for:

- meningitis

- arthritis

- both meningitis and arthritis

- neither

3. Other causes of brain symptoms are ...:

- *E. coli*, *S. hyicus* and salt intoxication

- *E. coli*, *S. hyicus* and *H. parasuis*

- E*. coli, H. parasuis* and salt intoxication

- *E. coli, M. hyosynoviae* and *S. hyicus*

4. Which serotype of *S. suis* is most frequently isolated in the Netherlands?

- 1

- 2

- 7

- 9

5. Which of the following items is not a preventive measure in the control of *S. suis*?

- When checking weaned piglets, work from young to old

- The piglet rearing pen is empty for at least 2 days after cleaning

- In the rearing pen, there is at least 1 drinking place for 10 piglets

- Sows are scab free

6. A criterion for instituting (partial) flock treatment is...?

- 5 percent of weaned piglets sick in 5 days

- 4 percent of weaned piglets sick in 4 days

- 5 percent of weaned piglets sick in 4 days

- 4 percent of weaned piglets sick in 5 days

7. Which statement(s) is/are correct? On an *S. suis* problem farm, there is:

- antibiotic resistance

- Defined Daily Dose Animal above the SDa norm

- use of second choice antibiotics when treating *S. suis*

- need for herd treatment

8. For what percentage of pigs may you leave second choice antibiotics on a farm?

- 5 percent

- 10 percent

- 15 percent

- 20 percent

9. What is the recommendation on the frequency of pathological examination in problem farms?

- 1x per year

- 2x per year

- 4x per year

- 6x per year

10. How long should a pig farmer keep treatment instructions from the veterinarian?

- 6 months

- 1 year

- 5 years

- 10 years
